# Supplementary material for: Identification of psychosocial problems in routine antenatal care in Ethiopia: A facility-based cross-sectional study
Source: Glob Ment Health (Camb). 2026 May 18;13:e111. doi: 10.1017/gmh.2026.10221 (PMC13231232; doi:10.1017/gmh.2026.10221)
Supplement: Catalao et al. supplementary material 1 — Catalao et al. supplementary material [file S2054425126102210sup001.docx]

**S1 File. Co-morbidity of psychosocial problems.**

1. Psychosocial conditions co-morbid with probable depression

|  | Probable depression (n=110) | Not depression  (n=1969) | χ^2^ (df)  p-value |
| --- | --- | --- | --- |
| Moderate to severe anxiety symptoms | 69 (62.7) | 258 (13.1) | 193.55 (1)  <0.001 |
| PTSD case | 25 (22.7) | 81 (4.1) | 74.60 (1)  <0.001 |
| Probable Intimate partner violence | 36 (32.7) | 253 (12.9) | 34.39 (1)  < 0.001 |
| Risky khat use | 7 (6.4) | 127 (6.5) | 0.0013 (1)  0.971 |
| Risky alcohol use | 1 (0.9) | 10 (0.5) |  |

1. Psychosocial conditions co-morbid with moderate to severe anxiety symptoms

|  | Moderate to severe anxiety symptoms (n=76) | Low anxiety symptoms  (n=2,003) | χ^2^ (df)  p-value |
| --- | --- | --- | --- |
| Probable depression | 32 (42.1) | 78 (3.9) | 213.35 (1)  <0.001 |
| PTSD case | 47 (61.8) | 59 (2.9) | 524.92 (1)  < 0.001 |
| Probable Intimate partner violence | 32 (42.1) | 257 (12.8) | 52.43 (1)  < 0.001 |
| Risky khat use | 10 (13.2) | 124 (6.2) | 5.90 (1)  =0.015 |
| Risky alcohol use | 1. (2.6)) | 9 (0.5) |  |

1. Psychosocial conditions co-morbid with probable PTSD

|  | Probable PTSD | No PTSD | χ^2^ (df)  p-value |
| --- | --- | --- | --- |
| Probable depression | 25 (23.6) | 85 (4.3) | 75.60 (1)  < 0.001 |
| Moderate to severe anxiety symptoms | 47 (44.3) | 29 (1.5) | 524.92 (1)  < 0.001 |
| Probable Intimate partner violence | 44 (41.5) | 245 (12.4) | 71.13 (1)  < 0.001 |
| Risky khat use | 13 (12.3) | 121 (6.1) | 6.27 (1)  < 0.012 |
| Risky alcohol use | 1 (0.9) | 10 (0.5) |  |

1. Psychosocial conditions co-morbid with probable intimate partner violence

|  | Probable IPV | No IPV | χ^2^ (df)  p-value |
| --- | --- | --- | --- |
| Probable depression | 36 (12.4) | 74 (4.1) | 34.39 (1)  <0.001 |
| Moderate to severe anxiety symptoms | 32 (11.1) | 257 (88.9) | 52.43 (1)  < 0.001 |
| Probable PTSD | 44 (15.2) | 62 (3.5) | 71.13 (1)  < 0.001 |
| Risky khat use | 33 (11.4) | 101 (5.6) | 13.77 (1)  < 0.001 |
| Risky alcohol use | 4 (1.4) | 1. (0.4) |  |

1. Psychosocial conditions co-morbid with risky khat use

|  | Risky khat use | Low risk/none | χ^2^ (df)  p-value |
| --- | --- | --- | --- |
| Probable depression | 7 (5.2) | 103 (5.3) | 0.0013 (1)  0.971 |
| Moderate to severe anxiety symptoms | 10 (7.5) | 66 (3.4) | 5.89 (1)  0.015 |
| Probable PTSD | 13 (9.7) | 93 (4.8) | 6.27 (1)  0.012 |
| Probable Intimate partner violence | 33 (24.6) | 256 (13.2) | 13.77 (1)  < 0.001 |
| Risky alcohol use | 1 (0.8) | 10 (0.5) |  |
