# Supplementary material for: Identification of psychosocial problems in routine antenatal care in Ethiopia: A facility-based cross-sectional study
Source: Glob Ment Health (Camb). 2026 May 18;13:e111. doi: 10.1017/gmh.2026.10221 (PMC13231232; doi:10.1017/gmh.2026.10221)
Supplement: Catalao et al. supplementary material 2 — Catalao et al. supplementary material [file S2054425126102210sup002.docx]

**S2 file. Clinical documentation by ANC staff and women´s experience of ANC worker questions.**

| **Clinical documentation by ANC staff** |  |  |
| --- | --- | --- |
| Psychosocial concern in presenting problem | Stress | 2 (0.1) |
| History of mental health problems | Documented, present | 1 (0.05) |
|  | Documented, none | 5 (0.2) |
|  | Not documented | 2073 (99.7) |
| History of substance use problems | Documented, present | 1 (0.05) |
|  | Documented, none | 34 (1.6) |
|  | Not documented | 2044 (98.3) |
| Current mental health problem |  | 1 (0.1) |
| Current substance use |  | 4 (0.2) |
| Current exposure to violence |  | 1 (0.1) |

| **Women’s experience of ANC worker questions** |  |  |
| --- | --- | --- |
| Asked how was feeling (emotionally) |  | 983 (47.3) |
| If asked, how comfortable about being asked about emotions (n=983) | Not at all | 2 (0.2) |
|  | Somewhat | 49 (5.0) |
|  | Comfortable | 932 (94.8) |
| If not, would have liked to have been asked (n=1096) |  | 640 (58.4) |
